# Glial Cell Activation and Oxidative Stress in Retinal Degeneration Induced by β-Alanine Caused Taurine Depletion and Light Exposure

**DOI:** 10.3390/ijms23010346

**Published:** 2021-12-29

**Authors:** Ana Martínez-Vacas, Johnny Di Pierdomenico, Francisco J. Valiente-Soriano, Manuel Vidal-Sanz, Serge Picaud, María Paz Villegas-Pérez, Diego García-Ayuso

**Affiliations:** 1 Departamento de Oftalmología, Facultad de Medicina, Instituto Murciano de Investigación Biosanitaria Hospital Virgen de la Arrixaca (IMIB-Virgen de la Arrixaca), Universidad de Murcia, 30120 Murcia, Spain; ana.m.v@um.es (A.M.-V.); johnnydp@um.es (J.D.P.); fjvaliente@um.es (F.J.V.-S.); manuel.vidal@um.es (M.V.-S.); 2 INSERM, CNRS, Institut de la Vision, Sorbonne Université, 75012 Paris, France; serge.picaud@inserm.fr

**Keywords:** taurine, β-alanine treated, retinal degeneration, phototoxicity, microglia, Müller cells, oxidative stress

## Abstract

We investigate glial cell activation and oxidative stress induced by taurine deficiency secondary to β-alanine administration and light exposure. Two months old Sprague-Dawley rats were divided into a control group and three experimental groups that were treated with 3% β-alanine in drinking water (taurine depleted) for two months, light exposed or both. Retinal and external thickness were measured in vivo at baseline and pre-processing with Spectral-Domain Optical Coherence Tomography (SD-OCT). Retinal cryostat cross sections were immunodetected with antibodies against various antigens to investigate microglial and macroglial cell reaction, photoreceptor outer segments, synaptic connections and oxidative stress. Taurine depletion caused a decrease in retinal thickness, shortening of photoreceptor outer segments, microglial cell activation, oxidative stress in the outer and inner nuclear layers and the ganglion cell layer and synaptic loss. These events were also observed in light exposed animals, which in addition showed photoreceptor death and macroglial cell reactivity. Light exposure under taurine depletion further increased glial cell reaction and oxidative stress. Finally, the retinal pigment epithelial cells were Fluorogold labeled and whole mounted, and we document that taurine depletion impairs their phagocytic capacity. We conclude that taurine depletion causes cell damage to various retinal layers including retinal pigment epithelial cells, photoreceptors and retinal ganglion cells, and increases the susceptibility of the photoreceptor outer segments to light damage. Thus, beta-alanine supplements should be used with caution.

## 1. Introduction

Taurine, a nonessential amino acid very abundant in many mammalian tissues, is highly concentrated in excitable tissues such as the retina [1]. Retinal taurine is collected and provided to photoreceptor cells by retinal pigment epithelial and Müller cells [2,3], and may be essential for neuronal survival in the retina [4,5] and for axonal transport in retinal ganglion cells [6,7] although it may have many more physiological roles that are still unclear [1,8]. Recent works have shown a relationship between taurine deficiency and retinal degeneration in humans [9,10]. Moreover, changes in taurine plasma levels are related to vigabatrin (an antiepileptic drug)-induced retinal toxicity in humans and rodents [11,12]. Thus, it appears that taurine is essential for retinal development and neuroprotection.

The exact mechanisms by which taurine acts in the retina to promote neuronal survival or neuroprotection remain unknown, especially in retinal degenerations, but in the central nervous system shows various protecting actions such as: antioxidation, restoration of anti-apoptotic protein expression, attenuation of endoplasmic reticulum stress or synaptic neuromodulation [2,10,13,14].

Taurine reduces oxidative stress [2], which plays a major role in several retinal degenerative diseases such as retinitis pigmentosa, age-related macular degeneration or diabetic retinopathy [2,15,16]. Oxidative stress and, in particular, reactive oxygen species have been related in the retina to pathogenic inflammation [2,17], an early sign of retinal degeneration [18,19,20,21]. Taurine is also known to participate in the regulation of retinal pigment epithelium phagocytosis [22] and is abundant in the subretinal deposits found in age related macular degeneration [23]. Interestingly, decreased taurine levels, together with increased levels of its analog β-alanine (see below), are one of the first signs of retinal degeneration in the Royal College of Surgeons (RCS) rat, an animal model of retinitis pigmentosa [24] that shows an impairment of the phagocytic capacity of the retinal pigment epithelium [25,26]. In RCS rats, photoreceptor degeneration courses with microglial cell activation and GFAP over-expression in Müller cells [19,20,27]. Moreover, micro and macroglial cell reaction are common features of inherited [19,20,28,29,30,31] and light-induced [21,32] photoreceptor degenerations [27,33]. This glial reactivity during photoreceptor degeneration may be related to retinal remodeling and be harmful to the retina [21,27,33]. Indeed, recent works have shown that the inhibition of microglial cells can reduce retinal degeneration [19,29,34,35].

Although some works have documented that taurine is a retinal neuroprotectant [11,36,37] necessary for retinal cell development [9,10] and survival [4,5,6], it remains to be shown whether decreased taurine plasma levels may trigger retinal degeneration, its pathways, and if it involves the retinal glial cells. In this work, we study in rats the macro and microglial cell changes in two of our experimental models of retinal degeneration: taurine depletion-induced [4,6] and light-induced [4,6,38], to investigate whether the noxious effects of taurine deficiency in the retina are exacerbated by light exposure.

## 2. Results

### 2.1. Taurine Plasma Levels

**Figure 1:**
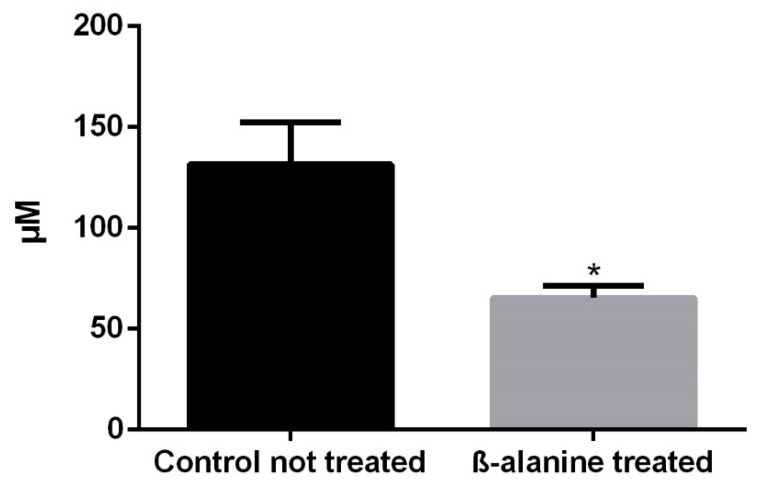
Taurine plasma levels in control and taurine depleted animals. Two months of treatment with β-alanine caused a significant reduction of the taurine plasma levels (*n* = 4, each subgroup). * = Statistically significant (*t*-test, *p* < 0.0001).

Plasma taurine levels were measured in four control and four β-alanine treated animals to verify the efficacy of the treatment. The treated animals had significantly lower plasma taurine levels than control non-treated animals (Figure 1; *t*-test *p* < 0.001).

### 2.2. SD-OCT

**Figure 2:**
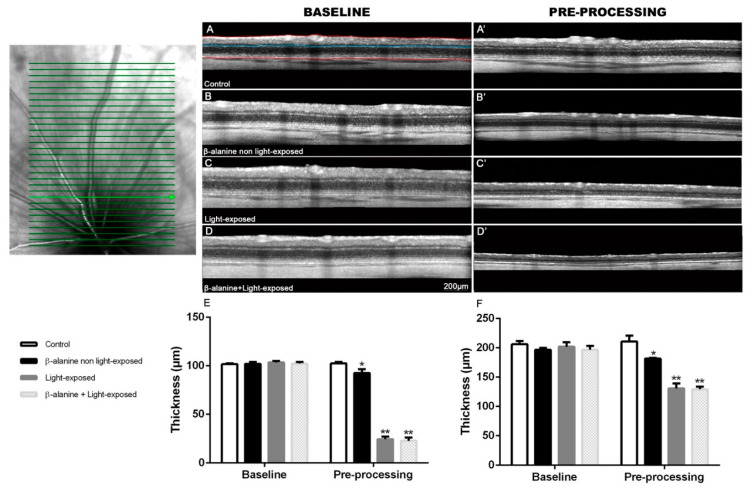
SD-OCT evaluation of total and outer retinal thickness. (**Upper Left**): Representative image of a scan image taken from a control animal that shows one of the cube scan protocols used: the dorsal. A similar ventral scan was also acquired in each animal. (**Upper right**): Representative SD-OCT scans obtained at baseline (**left column**) and pre-processing (**right column**) of same animals belonging to different subgroups: control (**A**,**A′**), β-alanine non light-exposed (**B**,**B′**), light-exposed (**C**,**C′**) and β-alanine and light-exposed (**D**,**D′**). In (**A**), the manually traced lines used for segmentation of the total retina are outlined in red and the line used for outer retina segmentation is outlined in blue just above the OPL. (**E**,**F**): Graphs showing mean ± SD outer retinal thickness (**E**) and mean ± SD total retinal thickness (**F**) in the different subgroups analyzed (**lower left**). * Significant difference when compared with pre-processing values of control animals *p* ≤ 0.0001. ** Significant difference when compared with pre-processing values of experimental group *p* ≤ 0.0001, Two-Way Anova test.

Mean total retinal thickness and outer retinal thickness were similar in all the subgroups at baseline (Table 1; Figure 2). At the pre-processing examination, total retinal thickness and outer retinal thickness were significantly decreased in the three experimental subgroups: β-alanine non light-exposed, light-exposed and β-alanine and light-exposed subgroups (Table 1; Figure 2). This decrease was larger in the β-alanine and light-exposed and in the light-exposed subgroups than in the β-alanine non light-exposed subgroup (Figure 2). Thus, the decrease in retinal thickness caused by light exposure was not augmented when photoexposed animals were treated with β-alanine (Figure 2, Table 1).

### 2.3. Outer Nuclear Layer Thickness, Photoreceptor Survival and Morphology

**Figure 3:**
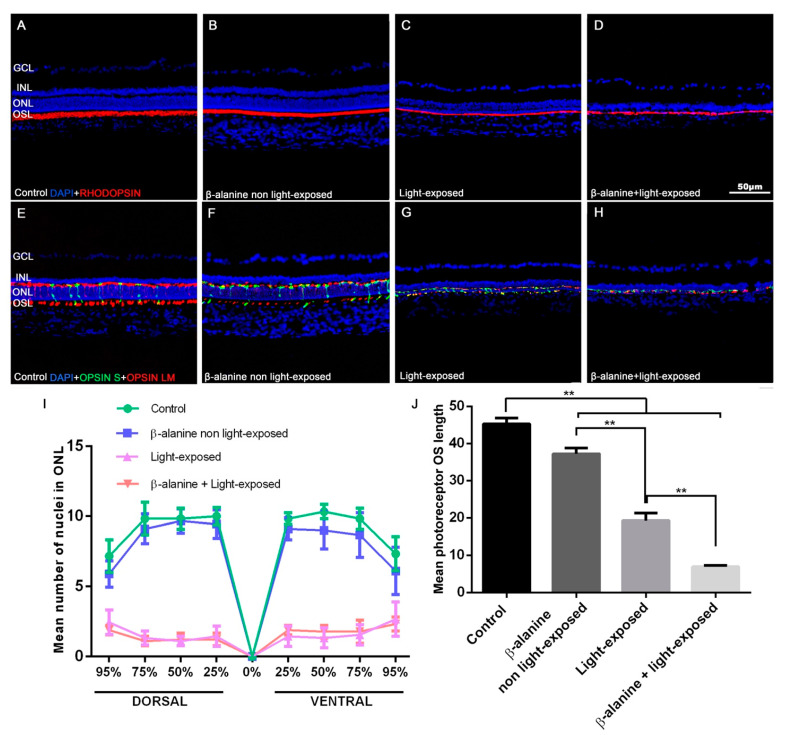
Photoreceptor outer segment morphology. Photomicrographs of retinal cross sections taken from representative animals of the different subgroups: control (**A**,**E**), β-alanine non light exposed (**B**,**F**), light-exposed (**C**,**G**) and β-alanine and light-exposed (**D**,**H**), showing rhodopsin (red, **A**–**D**), S (red, **E**–**H**) and L/M- (green, **E**–**H**) opsin immunoreactivity and nuclear DAPI counterstaining (blue, **A**–**H**). Rod and cone outer segments (OS) show reduced length in the experimental subgroups. The photoreceptor OS layer show reduced thickness in the experimental subgroups. Graphs showing mean numbers ± SD of ONL nuclei rows (**I**) and the mean thickness ± SD of OS layer (**J**) in the different subgroups. Scale bar: 50 µm. *n* = 10 per subgroup analyzed. ** Significant differences when compared to other subgroups (*p* ≤ 0.001). GCL: Ganglion cell layer. INL: Inner nuclear layer. ONL: Outer nuclear layer. OSL: Outer segments layer.

In control animals, outer nuclear layer (ONL) thickness ranged from 7 to 11 nuclei rows depending on the retinal area analyzed (Figure 3; Table 2), decreasing this number from the areas near the optic nerve to the retinal periphery. The thickness of the ONL in β-alanine non light-exposed animals was lower to that found in control animals specially in the ventral retina but we did not find significant differences between these subgroups in any of the areas analyzed (Figure 3; Table 2). However, light-exposed animals and β-alanine and light-exposed animals showed ONL thickness between one and three nuclei that were significantly lower in all the areas analyzed than those found in control and β-alanine non light-exposed animals. However, there were no significant differences in thickness between the β-alanine and light-exposed and the light-exposed subgroups in any of the areas analyzed (*p* = 0.9181; Figure 3).

The morphology of L/M- and S- cone outer segments (OS) and the thickness of the photoreceptor OS layer varied in the different groups. The photoreceptor OS layer was significantly thinner in the three experimental groups when compared to the control group (Figure 3). The β-alanine non light-exposed animals showed slightly, but significantly, thinner OS layer (Figure 3). The light-exposed animals and the β-alanine and light-exposed animals showed severely thinned OS layer, and this was more accentuated in the β-alanine and light-exposed group (Figure 3).

### 2.4. Microglial Cell Numbers

**Figure 4:**
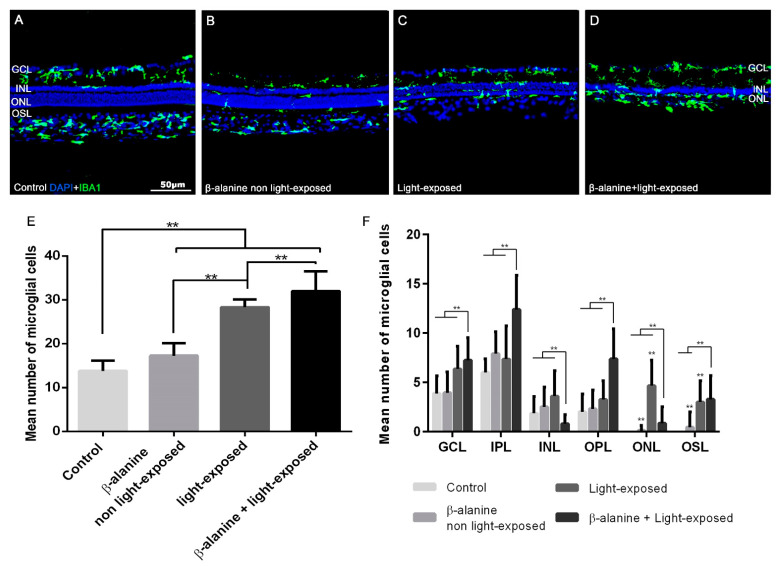
Microglial cell quantification. Upper part: Photomicrographs taken from retinal cross sections of representative animals from the different subgroups: control (**A**), β-alanine non light-exposed (**B**), light-exposed (**C**) and β-alanine and light-exposed (**D**) rats showing microglial cell immunoreactivity (green) and DAPI nuclear counterstaining (blue). Microglial cells can be seen in the outer retinal layers in the retinas of the experimental subgroups (**B**–**D**). Lower part: Bar graphs showing the mean ± SD numbers of microglial cells per retina (**E**) and per retinal layer (**F**) in the different experimental subgroups. In F, the bar for the control subgroup is missing in the ONL and OSL because the retinas of control animals did not show microglial cells in these layers. Scale bar: 50 µm. *n* = 10 per subgroup analyzed. In (**E**) we show ** Significant differences between the control subgroup and the experimental subgroups and between the different experimental subgroups. *p* ≤ 0.001 (*t*-test). In (**F**) we show ** Significant differences between the β-alanine and light-exposed subgroup and all the other subgroups in all the retinal layers except in the OSL, where this group showed significant differences only with the β-alanine non-light exposed group and light-exposed group *p* ≤ 0.001 (*t*-test). GCL: Ganglion cell layer. INL: Inner nuclear layer. ONL: Outer nuclear layer. OSL: Outer segments layer. ** *p* ≤ 0.0001 (*t*-test).

The mean numbers of Iba-1+ cells per section were 13.81 ± 2.33 in control animals, 17.29 ± 2.84 in β-alanine non light-exposed animals, 28.32 ± 1.8 in light-exposed animals and 31.97 ± 4.52 in β-alanine and light-exposed animals (Figure 4). In the experimental subgroups we observed morphological signs of microglial cell activation (retraction of processes and thickening of the processes and soma) (Figure 4). When we compared the numbers of microglial cells between the subgroups, we found that there were significant differences in the mean numbers of microglial cells between the control subgroup and all the experimental subgroups (Figure 4E). Moreover, there were also significant differences in the number of microglial cells between the β-alanine non light-exposed and the light-exposed subgroups (*p* < 0.001) (Figure 4E) and between the light-exposed and the β-alanine and light-exposed subgroup (*p* < 0.001) (Figure 4F). These results document that β-alanine treatment causes a significant increase in the total number of microglial cells in the retina that is incremented further with light exposure and with the combination of β-alanine and light exposure.

The numbers of microglial cells in the different retinal layers are shown in Table 3. In control animals, no Iba-1+ cells were found in the outermost retinal layers (ONL and photoreceptor OS layer; Figure 4F). All the other subgroups had microglial cells in the outer retinal layers (ONL and photoreceptor OS layer) and also showed increased numbers of microglial cells in the different retinal layers. When we compared the numbers of microglial cells in the different retinal layers between the different subgroups, we found that the β-alanine and light-exposed subgroup showed significantly increased numbers of microglial cells than all the other subgroups in all the retinal layers except in the INL and ONL layers, where the numbers of microglial cells were similar between all subgroups (Figure 4F), and in the OS layer where it showed significant differences only with the β-alanine non light-exposed subgroup but not with the light-exposed subgroup (Figure 4F). In addition, the light-exposed subgroup showed significantly more microglial cells in the ONL than the β-alanine not light-exposed and the β-alanine and light-exposed subgroups.

### 2.5. Macroglial Cell Reactivity

**Figure 5:**
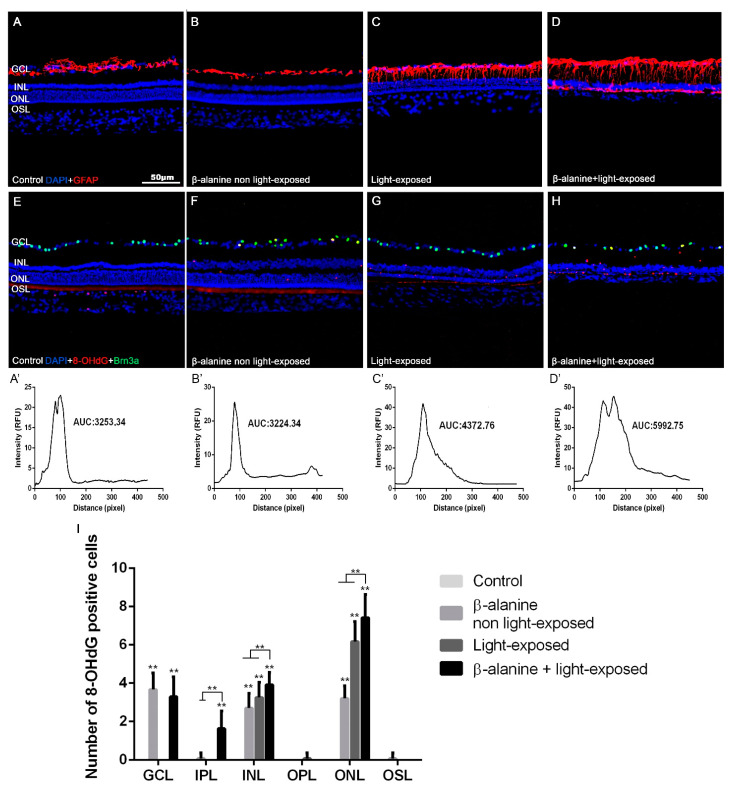
GFAP immunoreactivity and oxidative stress. Photomicrographs of retinal cross sections taken from representative animals of the different subgroups: control (**A**,**E**), β-alanine non light-exposed (**B**,**F**), light-exposed (**C**,**G**) and combined β-alanine and light-exposed (**D**,**H**) rats, showing GFAP (**A**–**D**, **red**) immunoreactivity, 8-OHdG+ cells and Brn3a+ cells (**red** and **green**, **E**–**H**) and DAPI nuclear counterstaining (**blue**, **A**–**H**). The GFAP immunoreactivity can be observed in astrocytes (**A**–**D**) and Müller cells (**B**–**D**), while the 8-OHdG+ immunoreactivity is observed in the photoreceptor outer segments in all subgroups (**E**–**H**), in some cells in the different retinal layers in the three experimental subgroups and, specifically, in retinal ganglion cells in both β-alanine treated groups. Graphs showing mean GFAP intensity in each experimental group and the area under the curve (AUC; **A’**–**D’**) and mean numbers of 8-OHdG positive cells per retinal layer (**I**). Scale bar: 50 µm. ** Significant differences when compared to other subgroups (*p* ≤ 0.001). GCL: Ganglion cell layer. IPL: Inner Plexiform Layer. INL: Inner nuclear layer. OPL: Outer Plexiform Layer. ONL: Outer nuclear layer. OSL: Outer segments layer.

In control non-treated animals, GFAP signal was observed only in the astrocytes of the GCL and nerve fiber layer (Figure 5A). In β-alanine non light-exposed animals, GFAP immunoreactivity was similar to that found in control animals (Figure 5B). However, in light-exposed animals, GFAP immunoreactivity was found not only in astrocytes but also in the inner processes of Müller cells (Figure 5C). Finally, in the β-alanine and light-exposed subgroup, GFAP immunoreactivity spread to both the inner and outer processes of the Müller cells, and therefore reached the more external retinal layers, up to the retinal pigment epithelium (Figure 5D).

Mean Relative Fluorescence Units (RFU) of GFAP immunoreactivity were 3314.37 ± 1012.24 (*n* = 8) in the control subgroup, 3296.67 ± 496.82 (*n* = 8) in the β-alanine non light-exposed subgroup, 4109.03 ± 213.08 (*n* = 8) in the light-exposed subgroup and 5577.21 ± 1183.55 (*n* = 8) in the β-alanine and light-exposed subgroup. When we compared these numbers, we found significant differences between the four different subgroups. Then we compared the subgroups two by two, and we found that RFU were significantly higher in the β-alanine and light-exposed subgroup when compared to the control subgroup (*p* = 0.0117; *t*-test), to the β-alanine non light-exposed subgroup (*p* = 0.0041; *t*-test) or to the light-exposed subgroup (*p* = 0.0258; *t*-test). This last subgroup showed also significantly higher RFU than the control subgroup (*p* = 0.0105; *t*-test) or the β-alanine non light-exposed subgroup (*p* = 0.0099; *t*-test). Therefore, light exposure and the combination of β-alanine and light exposure causes a significant increase of GFAP immunoreactivity.

### 2.6. Oxidative Stress

The photoreceptor outer segments were 8-OHdG immunoreactive in all the subgroups (Figure 5E–G). However, the number and distribution of the 8-OHdG immunoreactive cells differed between the subgroups. The retinas of control animals did not contain any 8-OHdG immunoreactive cells (Figure 5E). In the retinas of β-alanine non light-exposed animals we observed a few 8-OHdG+ cells situated in the ONL, inner nuclear layer (INL) and ganglion cell layer (GCL), (Figure 5F). The retinas of light-exposed animals showed more 8-OHdG+ cells than the previous subgroup, but they were situated only in the ONL, and INL (Figure 5G). Finally, in the retinas of β-alanine and light-exposed animals there were more 8-OHdG+ cells than in the previous subgroups and they were situated in the ONL, INL, inner plexiform layer (IPL) and GCL (Figure 5H). The 8-OHdG+ cells were particularly abundant in the remainder of the ONL in this last subgroup (Figure 5D). These results document oxidative damage to different retinal cells in the subgroups. It appears that all the experimental strategies caused oxidative damage to the photoreceptors and cells of the inner nuclear layer, but that only β-alanine administration caused oxidative damage to the retinal ganglion cells.

### 2.7. Synaptic Connections

**Figure 6:**
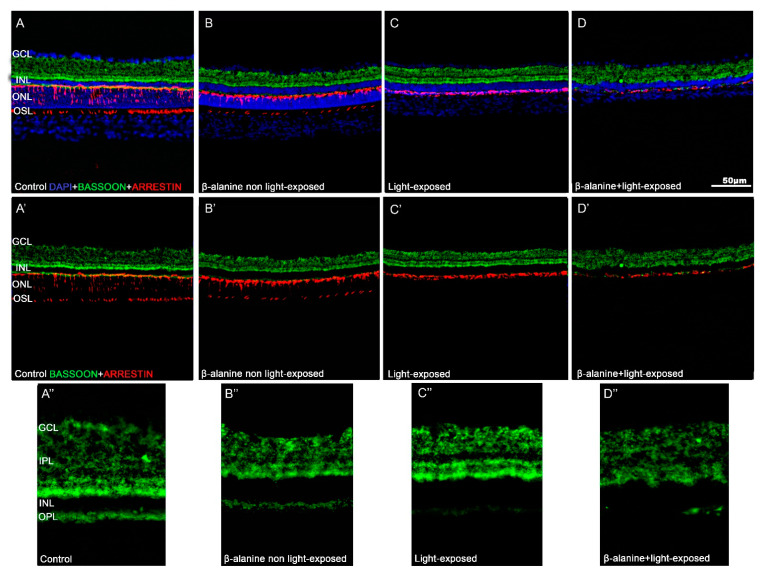
Bassoon immunoreactivity. Photomicrographs of retinal cross sections taken from representative animals of the different subgroups: Control (**A**,**A′**,**A**″), β-alanine non light-exposed (**B**,**B′**,**B**″), light-exposed (**C**,**C′**,**C**″), and combined β-alanine and light-exposed (**D**,**D′**,**D**″) rats showing bassoon (**green**) and arrestin (**red**) immunoreactivity. Figures (**A**–**D**) are the same shown in **A′**–**D′** but include DAPI counterstaining (**blue**). Figures **A″**–**D″** show high power magnifications of the microphotographs shown in **A′**–**D′**. Bar: 50 µm. GCL: Ganglion cell layer. IPL: Inner Plexiform layer. INL: Inner nuclear layer. OPL: Outer Plexiform layer. ONL: Outer nuclear layer. OSL: Outer segments layer.

In the retinas of control (naïve) animals, bassoon immunoreactivity was seen in both plexiform layers of the retina (Figure 6A,A′,A″). In the OPL this immunofluorescence was punctate and homogeneous, while in the IPL several immunofluorescent bands were observed (Figure 6A″). In the retinas of β-alanine non light-exposed animals, the plexiform layers were thinner and bassoon staining was thus less abundant both in the IPL and OPL, and although it maintained the morphology found in control animals it was less intense (Figure 6B,B′,B″). In the retinas of the light-exposed animals bassoon staining was intense and similar to that found in control animals in the IPL, although this layer was thinner, but it was very weak in the OPL (Figure 6C,C′,C″). Finally, in the retinas of β-alanine and light-exposed animals bassoon immunoreactivity could only be seen in small spots of the OPL and it was also less intense than that found in control animals in the IPL, where the typical bands found in the other experimental groups could not be clearly identified (Figure 6D,D′,D″).

### 2.8. Retinal Pigment Epithelium Function and Morphology

**Figure 7:**
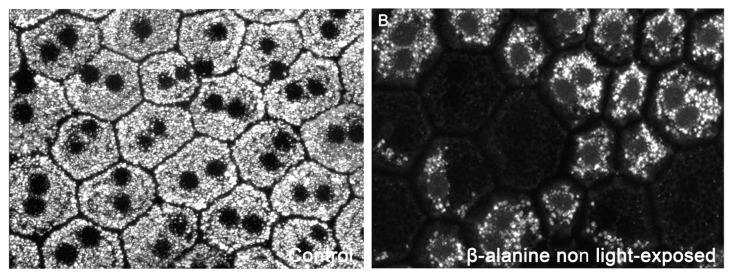
Photomicrographs showing the morphology of the retinal pigment epithelium in control (**A**) and taurine depleted (**B**) rats acquired with confocal microscopy. In taurine depleted animals some retinal pigment epithelial cells show less or do not show FG accumulations within the cytoplasm.

In the retinas of control (naïve) animals, Fluorogold (FG) accumulates homogeneously in the cytoplasm of the retinal pigment epithelium cells (Figure 7). In the retinas of β-alanine treated animals, we observed in all cells that there was less FG accumulation in the cytoplasm, and this was grossly exaggerated in some cells that showed FG only in a part of the cytoplasm only or did not show FG accumulations at all (Figure 7).

## 3. Discussion

One of the existing approaches to study the role of taurine in tissues such as the retina is to induce its systemic depletion using pharmacological treatments [1,4,5] and then studying the alterations triggered in the target tissue, i.e., the retina. It is well established that the administration of β-alanine on the drinking water of rats at a concentration of 3% causes a decline in taurine plasma levels [4,6,39,40,41,42] and loss of photoreceptors and retinal ganglion cells [27,43]. Moreover, light exposure has been shown to worsen the harmful effects of taurine depletion [4,6]. It is important to note that, although some studies have documented that the administration of small doses of β-alanine may have beneficial effects in different tissues [44,45], at high doses the results are toxic. The deleterious effect of β-alanine on the retina and other tissues occurs when it causes taurine depletion [46], which is achieved when administered to experimental animals at ≥3% concentration in the drinking water [46]. In this work, we have analyzed macro and microglial cells in the rat retina after taurine depletion, alone or combined with light exposure to examine their role in these types of retinal degeneration.

In this study we document that while control animals show normal retinal morphology, microglial cells in the GCL, IPL, INL and OPL, and GFAP immunoreactivity was only in astrocytes of the innermost retinal layers [18,20,47,48,49,50,51], taurine depletion, light exposure or the combination of both cause alterations of the numbers and distribution of microglial cells and GFAP immunoreactivity in Müller cells.

### 3.1. Photoreceptor Outer Segment Degeneration Caused by Taurine Depletion Is Exacerbated by Light Exposure

We show that β-alanine non light-exposed animals exhibit shortened outer segments of photoreceptors. This affectation could be the result of photoreceptor outer segment degradation [4,5,6,52,53] triggered by taurine deficiency. We show that taurine depletion causes oxidative stress to photoreceptors (see below). The shortening of the outer segment of photoreceptors was even more marked when β-alanine and light exposure were combined. These results are in accordance with previous works [4,5] and confirm that light exposure exacerbates the deleterious effects of taurine depletion on the photoreceptor outer segments. Taurine depletion alone did not appear to cause photoreceptor death, as not in this study nor in previous studies in the rodent retina [4,5,7,54] was significant thinning of the ONL found. However, this may not be completely true because it has been shown that taurine depletion ultimately causes cone death [4,55,56] which we may have not observed in this work because the rat retina is largely rod-dominated (cones represent only 0.85% of the retinal photoreceptors; [57] we have not carried out cone quantification in retinal whole mounts as in previous works [4,5].

In this study and in previous studies [4,38,58,59] light exposure causes photoreceptor death that is manifested by significant thinning of the ONL in comparison with control and β-alanine non light-exposed animals. This death is exacerbated when β-alanine treatment is combined with light exposure, in accordance with our previous study [4]. The experimental model of light-induced retinal degeneration used in this work causes rapid and progressive degeneration of both rods and cones, disruption of the photoreceptor mosaic and long-term alterations in all retinal layers, namely retinal remodeling, leading to retinal ganglion cell loss [27,38,43,60,61]. Some authors have proposed that taurine depletion-induced retinal degeneration is light dependent [3,5,16,62,63,64], and more recently we have confirmed that taurine depletion increases photoreceptor sensitivity to light [4]. Interestingly, a recent report suggests a role for taurine in the retinoids visual cycle [65] which could explain visual loss in vigabatrin treated patients [1,66,67,68] that could be thus the result of taurine depletion and light exposure [11,56,69]. Interestingly, it has also been documented that taurine may protect photoreceptors against light-induced retinal degeneration [14,70,71,72,73].

To determine whether taurine-depletion induced retinal degeneration was attributable to oxidative stress we used a marker for DNA oxidative damage 8-OHdG [74,75] and we analyzed qualitatively the number of 8-OHdG+ cells per retina in the different subgroups. We show that while both light exposure and β-alanine administration alone or in combination cause oxidative damage to photoreceptors and cells of the inner nuclear layer, which is more severe when the combination is used, only β-alanine administration, alone or in combination with light exposure causes oxidative damage to retinal ganglion cells. This is in accordance with our previous works showing that taurine depletion directly damages retinal ganglion cells in vigabatrin [56], guanidoethane sulfonate (GES) [5] and β-alanine [4,6] treated animals. The absence of staining in the light-exposed animals indicates that this retinal ganglion cell affectation is independent to photoreceptor loss, contrary to the secondary affectation of retinal ganglion cells reported previously by our group in inherited retinal degeneration [27,43,50,76,77,78]. Similarly, the retinal ganglion cell degeneration occurring in light-exposed animals was only observed many months after light exposure and is thus likely a secondary degeneration [27,38,43,60]. Moreover, we document using the bassoon antibody that labels synaptic ribbons in the OPL and conventional synapses in the IPL [79] that all the experimental strategies caused loss of ribbon synapses in the OPL, and that β-alanine administration also causes alterations of the IPL synapses both alone and when combined with light exposure. Therefore, we show that taurine depletion induces oxidative stress in the retina, causing neuronal death and thus loss of synaptic connections in the OPL and the IPL, suggesting that both inner and outer retinal cell death may be caused by taurine depletion.

To shed light on the mechanisms by which taurine depletion may be affecting the retina, we have used a labeling technique recently developed by our group to study the morphology and phagocytic capacity of the retinal pigment epithelium cells [75]. Using this technique, we document that, although in the retinas of β-alanine treated animals the hexagonal morphology of the retinal pigment epithelium is maintained, most of the cells showed less FG accumulation within their cytoplasm and some cells did not show FG accumulation at all. Since in this technique the retinal pigment epithelial cells become labeled through phagocytosis, this documents that taurine depletion impairs their phagocytic capacity. Thus, taurine depletion-induced photoreceptor outer segment degeneration could be caused, at least in part, by the impairment of the phagocytic capacity of the retinal pigment epithelium. Interestingly, we have documented similar alterations of FG accumulation in the retinal pigment epithelium cells in an animal model of inherited photoreceptor degeneration caused by an impairment of the phagocytic capacity, the RCS rat [75].

### 3.2. Glial Cell Activation Due to Taurine Depletion Is Exacerbated by Light Exposure

In the retinas of β-alanine non light-exposed animals we show morphological signs of microglial cell activation together with an increase in the number of microglial cells in the outer retinal layers. These events have been linked to early photoreceptor degeneration [20,31,58], which may be the trigger of microglial cell activation and proliferation [1,19,31,80,81,82,83].

Our results are in accordance with previous works reporting that light exposure induces microglial cell activation and migration to the outer retinal layers [21,27,31,32,43,58,61,84] and those suggesting that light exposure exacerbates taurine depletion-induced retinal degeneration [4,11]. Microglial cells migrate to the outer retinal layers probably due to the need to phagocytize the detritus of damaged/death cells [19,20,31,47]. The observed increase in microglial cells in the three outermost retinal layers together (OPL, ONL and OSL) was similar between the β-alanine non light-exposed and the β-alanine and light-exposed groups (data not shown). However, there were more microglial cells in the ONL in the light-exposed group and in the OPL in the β-alanine and light-exposed group, suggesting microglial migration to the most affected layers: while light exposure is more toxic to photoreceptor nuclei, taurine depletion is more toxic to photoreceptor outer segments and its synaptic connections. The increase in the numbers of Iba-1+ cells found in the outer retinal layers was not accompanied by a decrease in their numbers in the inner retina, suggesting microglial cell proliferation.

GFAP over expression in astrocytes and Müller cells is a sign of retinal inflammation [20,21,85,86] and an early sign of photoreceptor degeneration [20,21,87]. Β-alanine non light-exposed animals exhibit a stronger GFAP immunoreactivity, although restricted to astrocytes. Light exposure increases GFAP expression in astrocytes and Müller cells [21,58]. This expression increases even more when β-alanine is combined with light exposure, suggesting, again, that light exposure exacerbated the deleterious effects of taurine depletion or that taurine may protect from light damage [70,71,72,73].

## 4. Materials and Methods

### 4.1. Animal Handling

Two months old female albino Sprague-Dawley (SD) rats (*n* = 56) were obtained from the breeding colony of the University of Murcia. All animals were housed in temperature and light controlled rooms with a 12-h light/dark cycle (light from 8:00 a.m. to 8:00 p.m.) and had food and water *ad libitum*. Light intensity inside the cages ranged from 5 to 30 lux (scotopic and mesopic conditions). Animal manipulations were carried out in accordance with the Spanish and European Union regulations for the use of animals in research (Council Directive 86/609/EEC) and the ARVO Declaration for the use of animals in ophthalmic and vision research and were approved by the ethics committee of the University of Murcia. Appropriate measures were taken at all times to minimize pain or discomfort.

The animals were divided in two experimental groups (*n* = 28 each). In the first group, β-alanine (Sigma-Aldrich, Madrid, Spain) was administered in the drinking water while the second did not receive any treatment. One month after the beginning of the experiment, both groups were divided in two subgroups: one subgroup (*n* = 14) was exposed to light (see below) and the second (*n* = 14) was maintained under the normal light/dark cycle of our animal room (see previous paragraph). The treatment with β-alanine causes a decrease of the taurine plasma levels [4,6]. Thus, there were finally three experimental subgroups (β-alanine and light-exposed, β-alanine non light-exposed and light-exposed animals) and one control subgroup.

All animals were processed two months after the beginning of the experiment and also one month after light exposure in the two subgroups that were light-exposed. For sacrifice, the rats received a lethal dose of sodium pentobarbital (Dolethal, Vetoquinol, S.A., Lure, France) and were perfused transcardially through the ascending aorta, first with saline and then with 4% paraformaldehyde in 0.1 M phosphate buffer (PB; pH 7.4).

### 4.2. Light Exposure

Light exposure was carried out following the previously described protocols of our model of light-induced retinal degeneration [38,60]. Briefly, the animals were placed individually in transparent cages and exposed to cold white light (3000 lux; OSRAM GmbH, Munich, Germany) emitted from linear bulbs situated 20 cm above the cages continuously during 48 h. These animals were fed *ad libitum,* but the food was placed in Petri dishes at the bottom of the cage, to avoid light interference. In addition, to prevent the animals from burying their heads in the litter, a metal grid was placed at the bottom of the cages just above the litter.

### 4.3. Analysis of Taurine Plasma Levels

Blood samples were collected from the hearth animals just before euthanasia. The plasma was obtained by centrifugal separation and frozen at −20 °C until analysis for determination of taurine by chromatography. This was completed with an HPLC/MS system [4,6] consisting of an Agilent 1290 Infinity II Series HPLC (Agilent Technologies, Santa Clara, CA, USA) equipped with an Automated Multisampler module and a High Speed BinaryPump and connected to an Agilent 6550 Q-TOF Mass Spectrometer (Agilent Technologies, Santa Clara, CA, USA) using an Agilent Jet Stream Dual electrospray (AJS-Dual ESI) interface. Experimental parameters for HPLC and Q-TOF were set in MassHunter Workstation Data Acquisition software (Agilent Technologies, Santa Clara, CA, USA, Rev. B.08.00).

### 4.4. Spectral-Domain Optical Coherence Tomography

The retinas of all animals were explored in vivo at baseline (before β-alanine treatment) and two months later, just before processing using Spectral-Domain Optical Coherence Tomography (SD-OCT, Spectralis, Heidelberg Engineering, Heidelberg, Germany). For that purpose, rats were anesthetized with a mixture of ketamine (70 mg/kg Ketolar^®^, Pfizer, Alcobendas, Madrid, Spain) and xylazine (10 mg/kg Rompun^®^, Bayer, Kiel, Germany) and SD-OCT was performed using a contact lens specially designed for the rat eye was applied on the cornea [6,32,88], following previously described methods [6,32,88]. We used the “Fast” acquisition cube protocol that was set to perform a cube scan comprising 31 horizontal tomographic sections (512 A-scans) subtending 20° × 25° (width × height). Two non-overlapping cube scans were acquired per animal, one dorsal (Figure 2) and one ventral to the optic disk, so aligned that the lower and the upper lines of the dorsal and ventral scans respectively, were situated in the center of the optic disk. The follow up tool from the OCT program was used for the second (and last) examination, so that the exact same regions could be compared between consecutive exams.

In three lines of the scans (the superior and inferior lines of the dorsal and ventral scan, respectively, and the inferior line of the superior scan or the superior line of the ventral scan-central line-), we manually delineated the limits of the whole retina (from the nerve fiber layer to the retinal pigment epithelium) and of the outer retinal layers (from the OPL to the retinal pigment epithelium) and carried out an automatic segmentation of these layers. Next, using the caliper of the Spectralis software, we took four measurements of the total and outer retinal thicknesses in each of these three lines. In the superior and inferior lines, these measures were taken at four equidistant distances and, in the central line, because it comprises the optic disk, the measures were taken two at each side of the optic disk. Thus, we had 4 measures × 3 lines = 12 measures of total and outer thickness per animal.

### 4.5. Retinal Pigment Epithelium Labeling

Retinal pigment epithelium was labeled following previously described methods developed by our group [75]. Briefly, 1.5 µL of 3% FG (Fluorochrome Inc., Engelwood, CO, USA) diluted in saline was injected intravitreally through the superotemporal sclera using a Hamilton micro syringe (30 G; Hamilton 701 N, Esslab, Benfleet, UK) [29,75,86] The retinal pigment epithelium was dissected and flat mounted following previously described methods [75]) and photographed using a confocal microscope Leica SP8 (20×, 40× or 63×, Leica Microsystems, Wetzlar, Germany).

### 4.6. Tissue Processing and Immunohistofluorescence

After perfusion, the eyes were dissected maintaining their orientation. Cryoprotection was afforded by immersing the eyecups (without the cornea and the lens) first in phosphate-buffered saline (PBS) containing 15% sucrose for 1 day and later in PBS containing 30% sucrose for another day. Later, the eyecups were embedded in Tissue-tek and frozen at −80°C, and 16 microns thick vertical cryostat cross-sections containing the dorsal retina, the optic disk and the ventral retina were obtained.

All sections were subjected to a double immunohistofluorescence protocol following previously described procedures [20,28,38,50]. First, the sections were permeabilized by washing them in PBS + 0.5% Triton X-100 and then incubated overnight at 4 °C with a mixture of the primary antibodies (see next paragraph) diluted in blocking buffer solution (PBS, 2% Triton X-100, and 2% normal donkey serum; NDS, Jackson Immuno Research, Inc., Cambridge, UK). The next morning, the sections were incubated with a mixture of the secondary antibodies (see below) diluted in buffer solution (PBS and 2% Triton X-100) for 1 h at room temperature. Finally, sections were washed with PBS and mounted with an antifading mounting medium with DAPI (4′,6-diamidino-2-phenylindole; Vectashield Mounting Medium with DAPI, Vector Atom, Alicante, Spain).

#### 4.6.1. Primary Antibodies

Microglial cells were detected using a rabbit monoclonal anti-Iba1 antibody (1:1000–500; ab178846: Abcam, Cambridge, UK). Astrocytes and Müller cells were detected with a goat monoclonal anti- Glial fibrillary acidic protein (GFAP) antibody (1:500; 019-19741: Abcam, Cambridge, UK). The synaptic connections were detected with a mouse monoclonal anti-Bassoon antibody (1:750; ADI-VAM-PS003; Enzo life Science, Lausen, Switzerland). The L-cones, S-cones and rods outer segments were detected using a rabbit monoclonal anti-L/M-opsin (1:1200; ab5405; Chemicon-Millipore Iberica, Madrid, Spain), a goat monoclonal anti-S-opsin (1:1000; N-20; anti-OPN1SW; Santa Cruz Biotechnology, Heidelberg, Germany) and a mouse monoclonal anti-rhodopsin (1:1200, 1D4; Sigma-Aldrich, Madrid, Spain) antibody, respectively. Cone photoreceptors were detected using a rabbit monoclonal anti-cone arrestin antibody (1:1000; AB15282, Merck, Germany). Signs of Oxidative stress were detected with a monoclonal antibody against mouse α-8-hydroxy-2′-deoxyguanosine (8-OhdG; 1:1000, sc-66036; Santa Cruz Biotechnology, Heidelberg, Germany), which is a marker that binds oxidatively damaged proteins and DNA [75]. Retinal ganglion cells were detected using a goat polyclonal anti-Brn3a (1:750; C-20; Santa Cruz Biotechnology).

#### 4.6.2. Secondary Antibodies

Seven secondary antibodies were used to detect the different primary antibodies: donkey anti-goat Alexa Fluor 594 conjugate, donkey anti-goat Alexa Fluor 488 conjugate, donkey anti-mouse Alexa Fluor 594 conjugate, donkey anti-mouse Alexa Fluor 488 conjugate, donkey anti-rabbit Alexa Fluor 488 conjugate, donkey anti-rabbit Alexa Fluor 594 conjugate and goat anti-mouse Alexa Fluor 594 conjugate, (all diluted at 1:500; Molecular Probes, Invitrogen Inc., Madrid, Spain).

### 4.7. Image Analysis of Retinal Cross Sections and Quantification of Photoreceptors and Microglial Cells

Three vertical retinal cross sections spanning the optic disk were selected per animal and observed and photographed using a fluorescence microscope (Axioscop 2 Plus; Zeiss Mikroskopie, Jena, Germany) equipped with various filters and a digital high-resolution camera (ProgRes C10; Jenoptik, Jena, Germany).

The numbers of microglial cells were counted manually in each of these three sections. First, we counted the total numbers of microglial cells in the sections and later the numbers of microglial cells in various layers: the ganglion cell layer (GCL), inner plexiform layer (IPL), inner nuclear layer (INL), outer plexiform layer (OPL), outer nuclear layer (ONL) and outer segment layer (OSL) [20].

In each of the three sections selected per animal, eight photomicrographs (4 from the dorsal and 4 from the ventral retina) were acquired using 20× magnification (530 × 390 μm) at different standard distances between the optic nerve and the dorsal or ventral retinal periphery representing 25, 50, 75 and 95% of the length between the optic nerve and the retinal periphery. Images were further processed with Adobe Photoshop CS 6 (Adobe Systems, Inc., San Jose, CA, USA) when necessary.

In three representative regions of each of these photomicrographs, we counted manually the number of nuclei rows of the ONL. Thus, we obtained 3 measures per picture × 3 sections = 6 number of rows thickness measures for each retinal distance in every animal. [20]. The thickness of the photoreceptor OS layer was measured manually using ImageJ (National Institutes of Health, Bethesda, Maryland, USA; Available online: https://imagej.net/Welcome; accessed on 15 November 2021) in three representative regions of the photomicrographs taken at 50% and 95% of the length between the optic nerve and the retinal periphery.

Additional pictures were obtained for GFAP fluorescence quantification. These pictures were taken, eight per selected section as detailed before, but the gain and exposure time were fixed and corresponded to the mean automatic exposure time necessary to take these pictures in control animals. In these pictures, quantification of GFAP expression was carried out by obtaining the Relative Fluorescence Units (RFU) of the photographs using the tool “Histogram Analysis” of the Image Pro Plus software (IPP 5.1 for Windows; Media Cybernetics, Silver Spring, MD, USA) following previously described methods (Di Pierdomenico et al., 2020). We obtained for each picture a plot of the various GFAP fluorescence values and also their average and area under the curve (AUC), which indicates the total amount of fluorescence in the analyzed image.

### 4.8. Statistical Analysis

All quantitative data obtained are presented as the mean ± standard deviation (SD). The GraphPad Prism^®^ program (GraphPad Prism 6, GraphPad Software, LaJolla, CA, USA) was used for graph construction and statistical analysis. For comparisons of quantitative variables between two subgroups, we used the Student’s *t*-test and for comparisons between more than two subgroups the One-way-Anova test (and Tukey’s post hoc test). Differences were considered statistically significant when *p* ≤ 0.05.

## 5. Conclusions

In summary, our results document that taurine depletion induces glial cell activation and photoreceptor degeneration but also oxidative stress to retinal neurons and specifically to retinal ganglion cells and synaptic loss. We also document that these deleterious phenomena are exacerbated by light exposure and therefore that taurine depletion increases the susceptibility of the retina to phototoxic damage. Thus, caution should be exercised when administering β-alanine-based supplements, as an excess of recommended dose could lead to taurine depletion and cause retinal degeneration. It remains to be shown whether taurine supplementation may mitigate inflammation and be beneficial for the prevention of taurine deficiency or of retinal degenerative diseases caused or exacerbated by light-induced photoreceptor loss, such as inherited retinal degenerations or age-related macular degeneration.

## Figures and Tables

**Table 1:** SD-OCT retinal thickness measurements in control and experimental animals.

		Control	β-Alanine Non Light-Exposed	Light-Exposed	β-Alanine + Light-Exposed
OUTERRETINA	Baseline	101.6 ± 0.9 µm	102.1 ± 1.9 µm	101.6 ± 0.9 µm	102.1 ± 1.9 µm
	Pre-processing	102.4 ± 1.5 µm	92.5 ± 4.1 µm *^,†^	24.3 ± 2.6 µm *^,†^	22.7 ± 3.3 µm *^,†^
TOTAL RETINA	Baseline	206.2 ± 5.4 µm	196.7 ± 3.4 µm	201.9 ± 7.7 µm	196.8 ± 6.3 µm
	Pre-processing	210.8 ± 9.9 µm	181.6 ± 1.1 *^,†^	130.7 ± 8.5 µm *^,†^	129.3 ± 4.5 µm *^,†^

* Significant difference when compared pre-processing values with control animals; *p* ≤ 0.0001. ^†^ Significant difference when compared with baseline values of the same subgroup; *p* ≤ 0.0001.

**Table 2:** Number of nuclei rows in the outer nuclear layer in control and experimental animals.

	DORSAL				VENTRAL			
	95%	75%	50%	25%	25%	50%	75%	95%
Control	7.2 ± 1.2	9.8 ± 1.2	9.8 ± 0.8	10 ± 0.6	9.8 ± 0.4	10.3 ± 0.5	9.8 ± 0.8	7.3 ± 1.2
β-alanine non light-exposed	5.9 ± 0.9 *	9.1 ± 1	1.9 ± 0.9	9 ± 1	9.1 ± 0.8	9 ± 1.3 *	8.7 ± 1.6	6.1 ± 1.7
Light-exposed	2.4 ± 0.9 *^,†^	1.3 ± 0.5 *^,†^	1.1 ± 0.3 *^,†^	1 ± 0.7 *^,†^	1.4 ± 0.7 *^,†^	1.3 ± 0.7 *^,†^	1.6 ± 0.7 *^,†^	2.7 ± 1.2 *^,†^
β-alanine + Light exposed	1.9 ± 0.3 *^,†^	1.1 ± 0.3 *^,†^	1.2 ± 0.4 *^,†^	1.2 ± 0.4 *^,†^	1.9 ± 0.4 *^,†^	1.8 ± 0.4 *^,†^	1.8 ± 0.8 *^,†^	2.3 ± 0.5 *^,†^

* Significant difference when compared to the control group (*p* ≤ 0,05); ^†^ Significant differences when compared to the β-alanine non light-exposed subgroup (*p* ≤ 0.001).

**Table 3:** Number of microglial cells per layer and total in control and experimental animals.

	Control	β-Alanine Non Light-Exposed	Light-Exposed	β-Alanine + Light Exposed
GCL	3.9 ± 1.8	4 ± 2.1	6.4 ± 2.3 *	7.2 ± 2.3 *^,†^
IPL	6 ± 1.4	7.9 ± 2.2	7.4 ± 3.4	12.4 ± 3.5 *^,†,Ŧ^
INL	1.9 ± 1.7	2.5 ± 2	3.6 ± 2.6	0.8 ± 0.9 ^Ŧ^
OPL	2 ± 1.8	2.3 ± 1.9	3.3 ± 1.9	7.4 ± 3 *^,†,Ŧ^
ONL	0	0.1 ± 0.5	4.7 ± 2.6 *^,†^	0.8 ± 1.7 ^Ŧ^
OS	0	0.5 ± 1.5	3 ± 2.2 *^,†^	3.3 ± 2.4 *^,†^
All layers	13.8 ± 2.3	17.3 ± 2.8 *	28.3 ± 1.8 *^,†^	31.9 ± 4.5 *^,†,Ŧ^

* Statistical difference compared to the control subgroup (*p* ≤ 0.0001); ^†^ Statistical differences compared to the β-alanine non light-exposed subgroup (*p* ≤ 0.0001); ^Ŧ^ Statistical difference compared to Light-exposed subgroup (*p* ≤ 0.0001).

## Data Availability

Not applicable.
